# The impact of university STEM assets: A systematic review of the empirical evidence

**DOI:** 10.1371/journal.pone.0287005

**Published:** 2023-06-28

**Authors:** Chloe Billing, George Bramley, Carolin Ioramashvili, Robert Lynam, Magda Cepeda Zorrilla, Simon Collinson, Kelvin Humphreys, Konstantinos Kollydas, Fengjie Pan, Alice Pugh, Deniz Sevinc, Pei-Yu Yuan

**Affiliations:** 1 City-REDI, Birmingham Business School, University of Birmingham, Edgbaston, Birmingham, United Kingdom; 2 Department of Geography & Environment, London School of Economics and Political Science, London, United Kingdom; 3 Science Policy Research Unit (SPRU), Business School, University of Sussex, Brighton, United Kingdom; 4 Birmingham Business School, University of Birmingham, Edgbaston, Birmingham, United Kingdom; Alexandru Ioan Cuza University: Universitatea Alexandru Ioan Cuza, ROMANIA

## Abstract

**Background:**

Innovation ecosystems are an important driver of regional economic growth and development. STEM assets connected to universities may play an important role in such ecosystems.

**Objective:**

To systematically review the literature relating to the effect of university STEM assets on regional economies and innovation ecosystems, providing a better understanding of how the impact is generated and constrained, as well as identifying any gaps in knowledge.

**Methods:**

Keyword and text word searches using the Web of Science Core Collection (Clarivate), Econlit (EBSCO) and ERIC (EBSCO) were performed in July 2021 and February 2023. Papers were double screened on abstract and title, and were included if there was consensus that they fulfilled the inclusion criteria of: (i) relating to an OECD country; (ii) having been published between 1 January 2010 and 28 February 2023; and (iii) concerning the impact of STEM assets. Data extraction was undertaken for each article by a single reviewer and checked by a second reviewer. Due to the heterogeneity of the study designs and outcome measures used, it was not possible to perform a quantitative synthesis of results. A narrative synthesis was subsequently undertaken.

**Results:**

Of the 162 articles identified for detailed review, 34 were accepted as being sufficiently relevant to the study to be included for final analysis. Three important features identified were that the literature: i) is predominately concerned with supporting new businesses; ii) describes a high level of involvement with a university in providing that support; and iii studies economic impacts at local, regional and national levels.

**Discussion:**

The evidence points to a gap in the literature relating to looking at the broader impact of STEM assets and any corresponding transformational, system-level effects that go beyond narrowly defined, short to medium-term outcomes. The main limitation of this review is that information on STEM assets in the non-academic literature is not captured.

## Introduction

Innovation ecosystems are an important driver of regional growth and development [1]. They comprise all actors and organisations, generally at a local or regional level, involved in the innovation process. They include universities, private sector organisations conducting or commissioning research and development projects, third sector organisations such as charities, and public sector institutions such as research labs. It is a matter of intense scholarly debate whether successful ecosystems emerge and develop spontaneously, or whether they can be created and supported through policy intervention [2]. Answering this question is difficult due to the highly complex, interdependent and multifaceted nature of such systems.

In this paper, we report a systematic review of empirical evidence on the impacts of what we call STEM assets. For the purposes of this review, a STEM asset was defined as:

*‘A physical facility dedicated largely to the translation, development and transfer of scientific, technological or engineering innovation and knowledge and expertise which relates to new or improved business processes, products or services.’*

STEM assets include research labs, business parks, and business incubators. In particular, we focused on STEM assets that are associated with a university, e.g. based on a university campus, funded or run by or with a university, or collaborating closely with a university. These facilities may play an important role in innovation ecosystems by collectively acting as anchor institutions, creating a critical mass of researchers, bringing together different organisations within a region, such as universities and private businesses, setting priorities and research agendas, and developing and attracting skills [3].

Previous systematic reviews have focused on different aspects of the innovation policy landscape or specific components of innovation ecosystems. Sjöö and Hellström [4] focused on university-industry collaboration and the factors that stimulate these kinds of collaborations, including policy levers that can be applied. Similarly, Kanter [5] explored the links between different components in innovation ecosystems, including basic and applied research, small and large enterprises, and considered implications for business and employment growth. Jones, Meckel, and Taylor [6] reviewed the evidence on university incubators and entrepreneurship among graduates to develop a model of a student entrepreneur community of practice. Lander, Heinz, Hirschberg, and Stretch [7] conducted a systematic review of public involvement activities in biomedical research, drawing conclusions for bridging the gap between fundamental research and public policy and increasing public acceptance of research methods and the translation of new technologies. In a similar vein Feser [8], reviewed the literature on innovation intermediaries and knowledge sharing. Two recent reviews have focused on the relationship between universities and science and technology parks [9,10]. In contrast to these previous studies, the focus of the present paper is on the wider economic impacts of a diverse range of university STEM assets, including the conceptualisation and measurement of any such impacts.

The goal of this study is to provide an overview of what is already known in the academic literature about the impact of STEM assets on regional economies and innovation ecosystems, as well as to identify any gaps in knowledge. Overall, we find that the majority of studies focus on short-term impacts. The evidence points to a gap in the literature that takes a broader look at the impact of STEM assets, considering their systemic and transformational effects. More specifically this review aims to:

- Provide a better explanation of ‘how’ and ‘in what way’ STEM assets generate impact
- Provide more detail and granularity on the impact of STEM assets, in addition to commercial/economic impacts
- Identify the factors that constrain the innovation/productivity growth of these STEM assets and to what extent they are region or university specific.

The structure of the paper divides into three key sections. The next section introduces the methods used for the systematic review. The following section provides the results in narrative synthesis form. The final section discusses some limitations of the study and concludes.

## Methods

The review focuses on empirical studies across the fields of international business, economics, geography, and public policy. For comparability, we focus on empirical settings in OECD economies. From the studies included for final review, we extract information on the study methodology, the nature and intensity of university involvement, any inputs, activities, outputs and impacts measured by the study, as well the geographical extent of these impacts (local, regional, national, or supranational). We undertook a systematic search of the literature and a narrative synthesis [11,12].

### Inclusion and exclusion criteria for considering studies for this review

#### Types of studies

We did not screen studies by study design. We did, however, exclude commentaries and editorials, literature reviews that were not systematic reviews, conceptual and theoretical, and opinion pieces.

#### Type of interventions

We included studies that described physical STEM assets (incubators, science parks, accelerators, research parks, laboratories, testing centres, and innovation centres) or services provided by STEM assets (business support, technology transfer, training programmes, testing, information, diagnostics, and networking events such as conferences). We also considered studies that described programmes delivered by universities that support: innovation (product/process); knowledge exchange; cluster development, supply chain development; technical qualifications/degree level apprenticeships; and graduate programmes and internships where there was a possibility that they might draw on a STEM asset. We included studies that reported on businesses, entrepreneurs, innovators, intermediaries, or professional and business services working with STEM assets. We excluded studies where the participants were social enterprises and further education providers–unless working with a university or STEM asset–given their lack of focus on research.

#### Types of outcome measures

We included studies that provided information on at least one of the following outcome measures: innovation capability (process innovation, product innovation, or knowledge exchange) regional economic growth (start-ups, cluster development, or supply chains), business performance (turnover, productivity, profits), sustainability, inclusive growth, upskilling and employment, and environmental impact.

#### Geography

We included studies from OECD member economies only (comprising at the time of writing, Australia, Austria, Belgium, Canada, Chile, Colombia, Costa Rica, Czech Republic, Denmark, Estonia, Finland, France, Germany, Greece, Hungary, Iceland, Ireland, Israel, Italy, Japan, Korea, Latvia, Lithuania, Luxembourg, Mexico, Netherlands, New Zealand, Norway, Poland, Portugal, Slovak Republic, Slovenia, Spain, Sweden, Switzerland, Turkey, United Kingdom, United States). This decision was made so that studies included are from country contexts with comparable per capita incomes, as well as broadly democratic, capitalist governance structures and institutions.

### Search strategy

We performed a comprehensive search based on a combination of text words and controlled vocabulary search terms using the following databases: Web of Science Core Collection metasearch engine (Clarivate) which includes Science Citation Index Expanded (SCIE), Social Sciences Citation Index (SSCI), Arts & Humanities Citation Index (AHCI), Emerging Sources Citation Index (ESCI), Conference Proceedings Citation Index (CPCI), Book Citation Index (BKCI) and Current Chemical Reactions and Index Chemicus; Econlit (EBSCO) and Education Resources Information Center (ERIC) (EBSCO). The search strategy is attached in S1 File based on a variation of a PICOS (Population, Intervention, Comparator, Outcome and Study Design) statement that was the basis of the search terms and was used as the basis of the inclusion criteria.

The original searches for this review were performed between the 1^st^ and 27^th^ of July 2021 and included studies from January 2010 to July 2021. These searches were updated on 28^th^ February 2023 to include more recent studies. In addition, we identified further relevant articles cited in the selected texts. Searches were not limited by language or publication status. The results of searches were stored and managed in Endnote.

### Data collection and analysis

#### Selection of studies

The searches were undertaken by GB, CB and PY and exported into the Endnote database. Papers were double-screened on abstract and title and inclusion was based on consensus (AP, CB, DS, FP, KH, KK, PY, RL) with a third reviewer (CB, GB) adjudicating where necessary. At this stage, articles in languages other than English, French, German, Greek, Spanish, and Turkish were screened out, reflecting the language skills of the team. Articles that appeared to fulfil the inclusion criteria were retrieved. Full texts were screened and included texts were summarised in a standardised data extraction template. Data extraction was performed by a single reviewer and checked by a second reviewer (AP, CB, CI, DS, GB, FP, KH, KK, PY, RL) The data extraction template was designed by GB and based on the research questions, which were outlined by SC.

**Fig 1:**
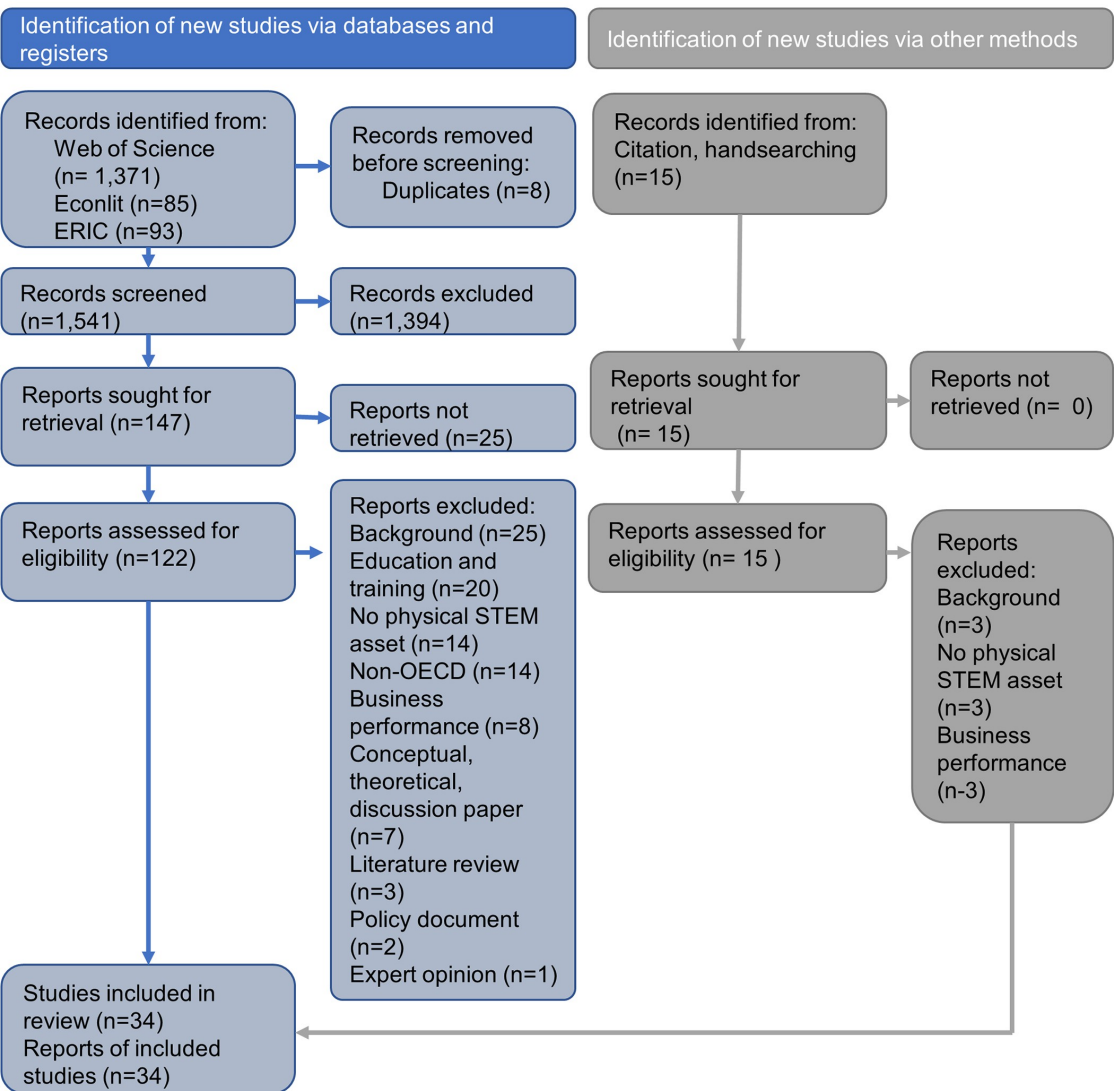
PRISMA chart summarising selection of papers.

The searches identified 1,556 articles, and from these 162 studies were independently selected by two reviewers based on titles and abstracts (see Fig 1). 128 studies were excluded from the 162 identified. An overview of excluded studies is provided in S1 Table. The most common reasons for excluding studies were that they describe education or training programmes without reference to a STEM asset (n = 20), there was no physical STEM asset (n = 17), or the case study was based in a non-OECD country (n = 14). We were unable to obtain 25 articles through online, local or national resources available to us.

#### Data extraction and analysis

For each text, the reviewers independently undertook data extraction using a piloted data extraction form. A blank form can be found in S2 File. Information was collated on the type of STEM asset (university, accelerator, incubator, science park, research park, fixed Infrastructure, labs, testing centre, innovation centre, collaboration, research centre, or other), university involvement, level of STEM asset autonomy and relationship with universities, geographical focus, resources, activities and outputs, performance metrics, available datasets on the asset, timeframe covered by the article, policy lessons derived in the article, and gaps in evidence. Due to the anticipated heterogeneity of study designs and reported outcomes, the extracted data is presented as a narrative synthesis and it was not possible to undertake a systematic assessment of risk of bias.

## Results

The characteristics of the 34 included studies are shown in S2 Table. Of the 147 articles identified for detailed review, 34 were accepted as being sufficiently relevant to the study. Of these 34 papers, all were published in English, and all, except for two which consisted of a simulation [13] and a systematic literature review [14], studied STEM assets in either North America (n = 15) or Europe (n = 17). The specific countries of focus included: the United States (n = 12), Spain (n = 3), the United Kingdom (n = 3), Canada (n = 2), France (n = 2), Ireland (n = 2), Sweden (n = 2), Turkey (n = 2), the Czech Republic (n = 1), Italy (n = 1), Mexico (n = 1), and Portugal (n = 1).

Table 1 provides an overview of the distribution of STEM assets and regions covered. Case studies from North America and the European Union are relatively evenly spread across different types of STEM assets. For both regions, as for the overall sample, incubators are the largest category with seven studies from the US and four studies from the EU. For both the UK and Turkey, the two countries not belonging to the two big regions, both incubators and universities are represented in the sample.

**Table 1:** Types of STEM assets and region of the case study.

	North America	European Union	UK	Turkey	No specific country	Total
Incubator	7	4	2	1		14
University	1	3	1	1	1	7
Innovation Centre	1	3				4
Accelerator	2				1	3
Labs	2					2
Research Centre	1	1				2
Research Park	1	1				2
Total	15	12	3	2	2	34

Note: Number of studies included in the review by type of STEM asset and region of the case study. North America includes Canada, Mexico and the USA.

### Study design of included papers

We classify the study design along two axes: qualitative vs quantitative, and by the number of observations included. Qualitative studies employ mostly qualitative methods, such as open-ended interviews and focus groups. Quantitative studies rely on statistical techniques to extract information from data. Mixed methods studies use both qualitative and quantitative methods. We also consider the number of subjects studied. We distinguish between case studies (one or a small number of cases that are studied in-depth, often comparatively), cross-sectional studies (a larger number of cases at a single point in time), and longitudinal research designs (one or more cases studied over several time periods). Of the papers included in our analysis, there was a mix of methodological approaches: qualitative studies were the most common (n = 14), followed by quantitative (n = 11), and mixed methods (n = 8). Of these approaches, the most frequently adopted study design was the case study (n = 19). Cross-sectional and longitudinal designs were equally as common, with 7 articles adopting each. In combination, the most popular research design was the qualitative case study (n = 8), followed by mixed methods case studies (n = 6).

### Overview of included papers

Papers were categorised in a mutually exclusive manner according to several factors of interest, the first of which being the type of STEM asset studied. In cases where papers studied multiple STEM assets, the one considered to be most central by the reviewer was selected. Incubators were the most frequently studied STEM asset, being the focus of almost half of the papers (n = 14), followed by universities themselves and their STEM-related functions such as knowledge transfer (n = 7). The remaining papers analysed innovation centres (n = 4), accelerators (n = 3), labs (n = 2), research/science parks (n = 2), and research centres (n = 2).

Each article was assigned a level of involvement with a university: low (n = 2), medium (n = 6), or high (n = 18). A high level of involvement was typically characterised by assets’ facilities/activities being located/undertaken on site, often involving students, or frequently delivered by the university. In contrast, papers coded as having a medium level of involvement described assets as being university-sponsored or affiliated and were not normally located on university premises. In several of the papers (n = 8), the level of involvement was either not clear or not sufficiently described to enable appropriate categorisation.

Finally, the geographical scale of the STEM assets’ impact was ascribed for each of the papers: local (n = 4), regional (n = 6), national (n = 13), and supranational (n = 1). Here, by local level, we mean the neighbourhood, town or city level, with the regional level going beyond a single city, and the national level meaning a whole nation-state. Geographical scales can be difficult to compare across countries with different administrative geographies and statistical classifications. Given the often vague and implicit descriptions of such impacts relating to different countries’ geographies, the highest level was selected for mutually-exclusive coding where impacts could be inferred as existing at multiple levels. The level of impact could not be identified in the remaining articles (n = 9).

#### Narrative synthesis

The following analysis is organised by type of STEM asset (where assets have been grouped according to the similarity of their functions), in each case identifying key findings relating to their university relationship, their inputs (resources and activities undertaken), the adopted methodological approach of the paper, and any measured impact.

#### Incubators and accelerators

Regarding the inputs for incubators and accelerators, it is possible to identify three broad groups of resources and activities which are apparent across the papers: facilities, funding, and support services [15–27]. Facilities refer to access to fixed infrastructure, such as office and manufacturing space, for businesses, graduates, or other entrepreneurs. Sources of funding vary, from local authorities to supranational authorities to private investments. Services typically include mentoring, business development workshops, skills training, academic expertise and knowledge acquisition, and networking, with the addition of curriculum content and extracurricular activities and experiences specific to graduates. Two of the articles focus specifically on pre-incubation [25,28], which has a much greater emphasis than incubators on activities that develop initial ideas into viable business ventures. Crisan et al identified three different levels of support provided by accelerators, ranging from limited to extended. They find that university accelerators usually offer limited support, with a focus on idea generation and validation [14].

Seven of the 14 incubator-focused studies [15,18,20,23,25–27], as well as one of the accelerator studies [29], were considered to have a high level of involvement with universities. Given that university business incubators are initiated by, and frequently located in or very close to, universities, this should not be surprising. However, in several instances, university engagement with the entrepreneurial team formation [23] and the operation of [25] their incubators were identified as having a significant role in shaping both incubator performance and the wider business ecosystem. Universities contribute to entrepreneurial teams in various ways, for example in board formation, or through softer interventions such as coaching and targeted educational offerings. Another study (although not focused on incubators) found that university-affiliated incubators worked with the most innovative firms which were thought to have the biggest growth potential [30].

The methodological approaches to the 16 studies were diverse, ranging from quantitative (n = 3), to qualitative (n = 6), to mixed method (n = 6) research strategies. The three purely quantitative articles differed in their specific approaches and research designs, which included statistical analysis informed by Structural Equation Modelling [28] and ANOVA analysis [22]. The quantitative elements of the mixed methods approaches included surveys [16], network analysis [19] and hypothesis testing using Pearson Chi-Square tests [23]. Among the qualitative and mixed method approaches, a case study design was frequently adopted [16–18,20,21,23,26,27,31], with interviews (typically semi-structured) being the most commonly used method [15,17,18,25,27,29,31]. However, these similar approaches were selected from differing theoretical frameworks, such as the absorptive capacity framework [27], a communications perspective [19], a more contextual Theory-based Impact Evaluation [17,18], and the Identity-Legitimacy-Life Cycle (ILLC) model [26].

Different methodological approaches adopted different measures for incubator and accelerator success. These ranged from the simple–such as employment and introduction of new products [29] or a relative count of new firms created across time [24]–to a wider range of 21 benchmark indicators [15]. As one study highlighted [25], the meaning of success to each individual incubator is likely to differ, ranging from the number or sustainability of firms established, to the quality of training and experience provided. Moreover, the IILC, which measures an incubator’s success by its ability to transition between its stages in development, necessitates differentiating between specific proxy measures used across time, transitioning from initial ad hoc monitoring to external standardised monitoring [26]. Follow-on funding secured by accelerated businesses may also be used as a performance indicator [14]. Thus, what is clear from the incubator and accelerator-related literature is that there is no one-size-fits-all measurement for evaluating participant success. Indeed, several different measures might potentially be required within the bounds of a single study, whether cross-sectional or longitudinal [25,26].

It is noteworthy that none of the studies focused their attention on measuring the broader impact of incubators and accelerators on the economy or society; they all focused on measuring the specific impact on the population of participating firms and incubatees. Positive commercial impacts were apparent across several studies, including increased firm survival rate, productivity, turnover growth, private investment, job creation, and new venture creation [17–19,21,22]. At the pre-incubation level, positive individual entrepreneurs’ perceptions of an incubator’s performance increased the desirability and confidence in starting and incubating a business [28], and another study evidenced incubatees’ continued improvement post-incubation [22]. One study–categorised as ‘university’ but which included analysis on incubators–found that incubation formed a condition for the most innovative young companies to achieve the highest growth rates [30]. A case study on the UBI Global-ranked top university-based tech incubator highlights the scale of the impact which such STEM assets can have, with the Digital Media Zone having helped over 400 companies, raised over $600 million, and created over 3,700 jobs since its inception [26]. The incubation literature also draws attention to non-commercial impacts such as providing professional development and learning opportunities for entrepreneurs, students, and university staff alike, raising the profile of the associated university and increasing connections with the wider community [20,26], as well as developing an entrepreneurial culture [14].

Surrogate entrepreneurship, whereby an experienced entrepreneur is added to a start-up’s senior leadership team (and the high level of university involvement it embodies) was identified as contributing to commercial benefits for incubatees [23]. But overall, the literature collectively explained the positive impacts in light of the inputs highlighted above: facilities, funding, and services involving human and/or social capital. Indeed, a lack of such inputs was identified by incubatees in one study as a constraint [25]. The social constructivist perspective of the Fisher ILLC model adopted in one paper explains incubator success as a function of its ability to gain differentially defined legitimacy from each new set of stakeholders it engaged with (and thus access to their respective resources needed to develop) [26].

#### University focused literature

Much like the papers discussing university business incubators, the seven articles categorised as ‘university’ highlighted the role of facilities, funding, and services involving human and/or social capital as key input resources and activities [30,32–34], which when not present were identified as constraining factors to innovation [35]. However, in this context, the university’s role as a mediator [36] can be understood as adding a fourth category of inputs which refers to its ‘third mission’ activities centred on business innovation. This category gives a prominent role to knowledge as a resource itself [30] and as the focus of entrepreneurial activities involving its transfer and diffusion, such as patenting and commercialisation, encouraging academic spinout companies, and coordinating collaboration between various stakeholders [35]. Such activities are typically conducted by technology transfer offices/centres [13,35], often as part of larger technoparks [33].

This subsection of the literature, then, acknowledges universities as prominently located spaces to generate and disseminate STEM knowledge, leading to local, regional, and national impacts such as organisational learning, business innovation, job creation, and economic growth [32,33,35,36]. However, it is equally as cautious in assigning them too much importance for generating such impacts. Indeed, not all universities have developed an entrepreneurial culture [30], and other factors, such as the availability of skills, have been identified as being more significant for regional growth [34]. Moreover, only two of the seven papers noted a high level of involvement between universities and the other relevant stakeholders discussed [33,35].

Methodologically, the university-focused literature was split fairly evenly: quantitative (n = 3), qualitative (n = 2), and mixed methods [13]. The two more simplistic quantitative designs involved administering surveys (one cross-sectional, one longitudinal) to businesses to understand their relationship with, and perceptions of, university activities [34,36]. The more advanced quantitative analytical techniques employed regression analysis, to identify contributing factors to spinoff generation as a way of measuring spillover [32]; fuzzy cognitive map modelling, to identify causal determinants of the success of technology transfer offices (TTOs) [13]; and crisp-set qualitative comparative analysis, to look for relationships between various support infrastructures and the growth of young innovative companies [30]. The latter used the change in employment level as a proxy for company growth, whilst the fuzzy cognitive map modelling measured TTO performance using the following proxies: number of spin-offs established, number of patents awarded, license income, income from industry-sponsored research contracts, and consulting income. One of the qualitative studies (both of which were case studies) also sought to understand the success of TTOs in promoting technology transfer but did so using semi-structured interviews with managers [33].

#### Innovation centres, labs, research centres and research parks

The remaining STEM assets are referred to as a group in this final section given i) the smaller number of papers which concern them (n = 10) and ii) that they share similar functions, as well as strong relationships with universities. It is notable that seven of these studies were categorised as having a high level of university involvement with the respective STEM asset, for which the two main reasons were specific location and their functional positionality as mediators between industrial firms and academia. Regarding the former, assets were typically based at, or within proximity to, the main university campus [37–40], whether they operated within a specific department [39,40] or almost independently of university management [41].

Regarding the specific inputs and activities undertaken in their mediating role, the STEM assets provide both concrete facilities and physical equipment, and a favourable environment for innovative collaboration between entrepreneurial firms as well as academic knowledge transfer to such firms [37,38,40–42]. Key to these innovation-related activities is hybrid researchers who work in the space directly between academia and industry [41], helping to close the gap which has typically been referred to as the ‘valley of death’. Not only is their specific academic knowledge particularly valued, but so too are the other components of researchers’ human capital, such as professional competencies, awareness of institutional culture, and accessible networks [37,41]. In one particular case, collaborative and interdisciplinary R&D, whilst supported by staff, is directed by university students, who both gain valuable skills and experience whilst contributing to highly sought-after innovative solutions to real industrial problems [39]. The environment and facilities provided by the STEM assets in another case also led to the development of linkages between community college students and local businesses through activities such as training, mentoring, and networking [37].

As well as knowledge transfer and innovation, assets frequently hold specific training programmes [41,43] and provide other services such as production process improvement and product testing [41]. One innovation centre provided assessments of business practices as well as technology clinics to assess needs and identify solutions to common issues [40]. In the case of research parks, supporting services also included food and drink, real estate and maintenance, which are provided by secondary organisations based on the site, making it an effective ‘micro cluster’ [38].

Funding for such assets is provided by governments at the regional, national, and supranational scale [41] as well as governmental agencies, membership fees from firms, donations, and spending by participants at in-person events and workshops [43]. However, the funding structure was also identified as an area of strain and even conflict with universities, as overhead resources for centre management, were difficult to source, and time for industry engagement was in conflict with academics’ other responsibilities, in particular developing academic publications [40,44].

The research approaches adopted by these ten studies were fairly evenly split. The two papers relating to labs [37,39] both opted for qualitative case study designs. Three studies on innovation centres adopted qualitative approaches, conducting semi-structured interviews with individuals working at the centres [40,41,44]. One of these researched an interdisciplinary approach between STEM and social sciences. In this case, the goal of the innovation centre was to simultaneously improve productivity as well as employment quality at supported businesses, and the paper documented the implementation from management and employee perspectives using repeated semi-structured interviews [44]. In contrast, another paper concerning innovation centres used a quantitative approach involving survey data and multiple linear regression techniques to better understand the factors affecting company turnover growth [45]. The remaining four papers, relating to research centres and research parks, all used quantitative methods. One of these also looked at identifying the factors, such as the number of patents and finance, which best predict the growth of companies based at research parks, but used the Mann-Whitney statistic method [42]. The final three articles sought to measure the wider impact of their respective STEM assets, but the impact was measured differently in each case. The research park study estimated its indirect and induced employment, differentiating between ‘core’ and ‘supporting’ firms based on site [38], whilst the research centre studies tried to estimate its regional and national economic impacts [43,46]. In one case, a proxy measure of regional economic impact was estimated by combining direct and indirect centre expenditures (generated using a regional input-output model) with many other additional impacts including income relating to licensing and intellectual property, taxable income from start-up firms which originated at the centre, and the cost savings for firms who hired graduates of the centre. To estimate the national economic impact, the regional proxy was complemented with a consumer surplus model to accommodate for spillover effects from which the public benefits [43]. Finally, one paper used administrative data and a double-matching approach to identify two control groups and causally estimate regional spillover effects from the research centre. While they found a positive short-term effect on employment, particularly employment of managers, there were no significant long-term effects of the STEM asset discernible on the regional economy [46].

Turning directly to the evaluation of the impacts generated by innovation centres, labs, research centres and research parks, several insightful points arise from these studies. Firstly, whilst the activities of these STEM assets and their positionality vis-à-vis academia and industry outlined above seemed suggestive of a direct relationship between existence and commercial impact, the effect is somewhat more nuanced, especially regarding innovation centres. Indeed, both studies conclude that interactions between the centres and firms alone are insufficient to explain economic impacts such as growth and increased employment. However, when combined with other factors–such as public financial support and other support infrastructures–such interactions become statistically significant [41,45].

Secondly, quantitative studies, whilst beneficial, are also problematic in several ways. One research centre study highlighted the problem of time lag, with the uptake and application of R&D and tangible indicators of other economic impacts taking a number of years to evidence before they could be measured and assessed [43]. Moreover, and of particular pertinence to this systematic review, the same paper also concluded that only using quantifiable measures considerably underestimates the scale of impact that STEM assets generate. Indeed, benefits can be unintended and unanticipated, such as providing local employers and community college students the space for training, as one of the lab studies documents [37]. Furthermore, quantitative studies are less able to effectively measure certain benefits such as improved reputation [37,42].

This links with the final point that the literature calls to attention: the non-commercial impacts generated by STEM assets. Not only can STEM assets positively affect regional and national employment [38,43], but society can benefit in a multitude of ways from the practical application of innovations in a wide breadth of sectors, from health care to education, to the environment [39]. Furthermore, interdisciplinary approaches that bring together social scientists, natural scientists and engineers may produce social impacts, such as improved job quality [44]. Similarly, multiple stakeholders can benefit from the activities which assets undertake, from the experience, skills and training developed by students and young career researchers in the short term to the businesses and firms who come to benefit from such human capital in the long term [37,43].

## Discussion, limitations, and conclusion

From the initial categorisation of studies, three important trends are apparent in the literature concerned with university STEM assets: 1) there is a significant focus on supporting new businesses, 2) assets typically have a high level of involvement with a university in providing that support and 3) economic impact is experienced and studied at local, regional and national levels. However, in particular, these wider impacts are not generally explored in detail, with a focus on more immediate, short- to medium-term outcomes. In the case of quantitative studies, the measured outcomes are mostly limited to easily quantifiable variables such as businesses supported or jobs created. In the case of qualitative studies, participants tend to be limited to direct beneficiaries of a programme, rather than including the views of a broader group of stakeholders.

For all types of STEM assets, inputs (facilities, funding, and services) were cited as key factors for the success of the intervention. In the case of universities and their translation activities, the mediating factor of the university forging connections between different actors play an important role and can be understood as another important factor. This highlights the importance of immaterial inputs, such as staff time invested in building relationships. Similarly, the evidence on innovation and centres, labs and research parts also points to the importance of moderating factors in achieving positive economic impact, including public financial support and other support infrastructures.

Corresponding to the diversity of inputs and their interaction, STEM assets also have a multitude of outputs and impacts in different domains. This is an issue in particular quantitative studies, which suffer from difficulties in measuring all of these. The gaps can be placed into two categories: unmeasured and non-commercial impacts. On the one hand, some positive impacts may not be measured. For example, an association with a university may be a quality signal for a young business, making it more likely to attract customers. This impact channel would be difficult to identify or quantify. On the other hand, some impacts are non-commercial, such as social and environmental benefits. In combination, then, these insights demonstrate the multitude of different proxy variables used to estimate impact, which can be particularly problematic for trying to achieve an all-encompassing, objective measure. This lack of consensus in the literature about how to measure impact most effectively was highlighted over a decade ago [43] and appears to remain as unresolved.

Few of the studies included here take wider, regional or macroeconomic impacts into account. This is somewhat surprising, as the main rationale for investing in such institutions is often to stimulate overall economic growth. While these economic impacts should generally be easily quantifiable, causal attribution of any impacts to a specific STEM asset is challenging given the multitude of factors involved in innovation ecosystems as well as the consideration of lag. Nonetheless, this represents a big gap in the literature which would benefit from further research. The present review has not taken into account non-academic literature, such as policy reports. As this subject is of acute policy interest, potentially valuable contributions, such as evaluation studies outside the academic literature, were out of scope for this systematic review.

Given the wide range of interventions, contexts, research questions and methodological approaches uncovered in the literature, it was not possible to conduct either a realist synthesis [47] to understand the influence of context on outcomes generated by STEM assets or a meta-analysis to understand average effect sizes of different types of STEM assets on economic performance. This calls for further research into the effects of STEM assets, in particular on a regional level. In the absence of a natural policy experiment, causal evidence on a wide range of assets will be challenging to present. Nonetheless, a comparative analysis of a smaller number of interventions would be feasible. As discussed, not all impacts will be readily quantifiable, pointing to the relevance of a mixed-methods approach in such studies. Moreover, interviews, as undertaken in a number of studies reviewed here, can shed more light on channels and mechanisms creating impact, than purely quantitative work.

While several systematic reviews study specific types of STEM assets [e.g., [8–10,14]], the present study is the first to consider different types of STEM assets jointly. While different types of organisations differ in their inputs, activities and organisational structure, the findings in this paper point to shared goals and ambitions, such as increasing employment, productivity and wider social goals. Such commonalities would call for studying these jointly and assessing how different STEM assets complement each other, or possibly duplicate certain functions in local economies. There is a gap in the literature studying the function of innovation intermediation and how this is performed at the local, regional or national level, looking across specific organisational forms such as research centres or incubators.

However, the present study also reveals the challenge such a unified treatment of STEM assets poses. Developing a general framework for STEM assets’ inputs, activities, outputs and impacts, such as developed by Crisan et al for the case of accelerators was not feasible due to the shear variation demonstrated between different types of STEM assets [14].

For policy makers, this implies a wide range of tools at their disposal to improve the translation of research findings into economic outcomes. Universities, as highly-involved partners in most of the STEM assets studies, play an important role in fostering these connections. However, they could also play a further role in coordinating different efforts. To do so, resources need to be in place to facilitate this coordination role. As several studies highlight, financial and managerial resources are not always available to the required extent. Providing sufficient resources would underline the strategic importance of translation activities, beyond an extension of academic research. Some studies highlighted the conflicting incentives academics face in the trading of time for academic research against engaging with industry. Aligning these incentives may prove crucial to strengthen the role of STEM assets, and more research is required to identify best practices in this respect.

## Supporting information

S1 Checklist PRISMA 2020 checklist. (DOCX)

S1 Table Data extraction sheet. (DOCX)

S2 Table Overview of included papers. (DOCX)

S1 File Search strategy. (DOCX)

S2 File Excluded papers. (DOCX)
