# Supplementary material for: The impact of university STEM assets: A systematic review of the empirical evidence
Source: PLoS One. 2023 Jun 28;18(6):e0287005. doi: 10.1371/journal.pone.0287005 (PMC10306183; doi:10.1371/journal.pone.0287005)
Supplement: S1 Table — (DOCX) [file pone.0287005.s002.docx]

# **Supplement 3**

**Data Extraction Sheet**

| **If Reject at Full Text – Provide a Principle Reason for Rejection** | | | |
| --- | --- | --- | --- |
| **Country of Case Study** |  | **Author date** |  |
| **ID number** |  | **Reference (Harvard Reference)** |  |
| **Title** |  | | |
| **Abstract** | *Copy and paste* | | |
| **Type of STEM asset** | University* |  | **University relationship with STEM asset – level of autonomy from University, how it has evolved and how many ideas currently flow through the University?** |
|  | Accelerators |  |  |
|  | Incubators |  |  |
|  | Science Parks |  |  |
|  | Research parks |  |  |
|  | Fixed Infrastructure |  |  |
|  | Labs |  |  |
|  | Testing centres |  |  |
|  | Research Centres |  |  |
|  | Innovation Centre |  |  |
|  | Collaboration |  |  |
|  | Other: |  |  |
| **University Involvement?** | Low |  |  |
|  | Medium |  |  |
|  | High |  |  |
| **Local / Regional / National / Supernational (e.g. European level) impact?** | |  | |
| **List of Inputs: what resources go into a STEM asset? (For this stage, some example indicators include: public investment received, expertise, facilities, and commercial income).** | | **List of Activities and outputs: what activities does the STEM asset undertake? (business capability development, events to engage with academics and businesses, and collaborative R&D projects).** | |
| **List of Intermediate outcomes: short-term and immediate changes or benefits from STEM asset activities (these are benefits that occur relatively quicker for users of the assets, i.e., in the first five to years)** | | **List of Later stage outcomes: longer-term changes or benefits from STEM asset activities (These can be intended longer term outcomes for recently established as well as reported outcomes recorded for well-established STEM assets)** | |
| **WHAT PROXY MEASURES / METRICS ARE BEING USED TO MEASURE THE IMPACT OF FOR THE STEM ASSET DESCRIBED IN THE TEXT– perhaps a methodological approach we could apply to our own research?** | | | |
| **Examples of Impact Indicators uses (Details on where they found the data / methodology) –**  **• Examples include: ‘Innovation Capability’, Growth, Turnover, Productivity, Sustainability, Inclusivity, Upskilling, Employment, Social inclusion, GVA, turnover, Economic impact, Social impact, Environmental impact**   \| WHAT IS BEING MEASURED \| PROXY/ PERFORMANCE METRIC \| DATA PROVIDED – DATE AND FIGURES PROVIDED \| \| --- \| --- \| --- \| \|  \|  \|  \| \|  \|  \|  \| \|  \|  \|  \| \|  \|  \|  \| | | | |
| **Available datasets** | | | |
| **Time frame of research data** | | | |
| **Lessons from and gaps in the current reports and academic literature** | | | |
| **Any general comments (further information on case studies)** | | | |
| **Key references cited** | | | |
| **Key website found related to the STEM asset** | | | |
| **Notes** | | | |
