# Supplementary material for: The impact of university STEM assets: A systematic review of the empirical evidence
Source: PLoS One. 2023 Jun 28;18(6):e0287005. doi: 10.1371/journal.pone.0287005 (PMC10306183; doi:10.1371/journal.pone.0287005)
Supplement: S2 Table — (DOCX) [file pone.0287005.s003.docx]

# **SUPPLEMENT 4**

**Overview of papers included in final review**

| **Author** | **STEM asset** | **Country** | **University Involvement** | **Study Design** |
| --- | --- | --- | --- | --- |
| Al-Dajani, H., et al., Graduate Entrepreneurship Incubation Environments: A Framework of Key Success Factors. Industry and Higher Education, 2014. 28(3): p. 201-213. | Incubators | UK | High | Qualitative cross-sectional |
| Al-Mubaraki, H.M., A.H. Muhammad, and M. Busler, Categories of incubator success: a case study of three New York incubator programmes. World Journal of Science Technology and Sustainable Development, 2015. 12(1): p. 2-12. | Incubators | US | Unclear | Qualitative case study |
| Audretsch, D.B., T.T. Aldridge, and M. Sanders, Social capital building and new business formation: A case study in Silicon Valley. International Small Business Journal-Researching Entrepreneurship, 2011. 29(2): p. 152-169. | Accelerators | US | Unclear | Mixed methods case study |
| Bigliardi, B., et al., EVALUATING THE PERFORMANCE OF TECHNOLOGY TRANSFER OFFICES: A FUZZY COGNITIVE MAPS APPROACH. 5th International Conference of Education, Research and Innovation, ed. L.G. Chova, A.L. Martinez, and I.C. Torres. 2012. 4801-4810. | University | n/a (simulation) | Unclear | Mixed methods case study |
| Breznitz, S.M. and Q. Zhang, Fostering the Growth of Student Start-Ups from University Accelerators: An Entrepreneurial Ecosystem Perspective. Industrial and Corporate Change, 2019. 28(4): p. 855-873. | Accelerators | Canada | High | Qualitative cross-sectional |
| Buckley, A.P. and S. Davis, Evaluating the Contribution of Technology Start-up Incubators: Exploring Methodological and Data-related Conundrums, in Proceedings of the 15th European Conference on Research Methodology for Business and Management Studies, V. Benson and F. Filippaios, Editors. 2016. p. 68-75. | Incubators | Ireland | Unclear | Mixed methods case study |
| Buckley, A.P. and S. Davis, The Contribution of Higher Education-Based Technology Start-Up Incubators to the Co-Production of Knowledge, Innovation and Growth: Experiences from the Edge. Industry and Higher Education, 2018. 32(4): p. 253-268. | Incubators | Ireland | High | Mixed methods case study |
| Ciuchta, M.P., et al., Imprinting and the progeny of university spin-offs. Journal of Technology Transfer, 2016. 41(5): p. 1113-1134. | University | US | Unclear | Quantitative longitudinal |
| Cooper, C.E., S.A. Hamel, and S.L. Connaughton, Motivations and Obstacles to Networking in a University Business Incubator. Journal of Technology Transfer, 2012. 37(4): p. 433-453. | Incubators | US | Medium | Mixed methods case study |
| Cormia, R.D., et al., Integrating Electron Microscopy into Nanoscience and Materials Engineering Programs, in Scanning Microscopies 2014, M.T. Postek, et al., Editors. 2014. | Labs | US | High | Qualitative case study |
| Covelli, B.J., et al., Forming a University-Based Business Incubator for Student and Community Entrepreneurs: A Case Study. Journal of Continuing Higher Education, 2020. 68(2): p. 117-127. | Incubators | US | High | Qualitative case study |
| Crișan EL, Salanță II, Beleiu IN, Bordean ON, Bunduchi R. A systematic literature review on accelerators. The Journal of Technology Transfer. 2021 Feb;46:62-89. | Accelerators | Global | Mixed | Literature review |
| Ferreira, J.J. and C. Fernandes, Cooperation between KIBS and Universities: An Empirical Study. Ifkad - Kcws 2012: 7th International Forum on Knowledge Asset Dynamics, 5th Knowledge Cities World Summit: Knowledge, Innovation and Sustainability: Integrating Micro & Macro Perspectives, ed. G. Schiuma, J.C. Spender, and T. Yigitcanlar. 2012. 1377-1393. | University | Portugal | Medium | Quantitative cross-sectional |
| Fett, P., The Science of Growth. Community College Journal, 2010. 80(3): p. 26-29. | Incubators | US | Medium | Qualitative case study |
| Fotso R. Evaluating the indirect effects of cluster-based innovation policies: the case of the Technological Research Institutes in France. The Journal of Technology Transfer. 2022 Aug;47(4):1070-114. | Research Centre | France | Low | Quantitative longitudinal |
| Gibson, L.J., J. Lim, and V. Pavlakovich-Kochi, The University Research Park as a Micro-cluster: Mapping Its Development and Anatomy. Studies in Regional Science, 2013. 43(2): p. 177-189. | Research parks | US | Unclear | Quantitative case study |
| Giordano Martinez, K.R., A. Fernandez-Laviada, and A. Herrero Crespo, Influence of Business Incubators Performance on Entrepreneurial Intentions and Its Antecedents during the Pre-incubation Stage. Entrepreneurship Research Journal, 2018. 8(2). | Incubators | Mexico | Unclear | Quantitative cross-sectional |
| Holmström J, Magnusson J, Mähring M. Orchestrating digital innovation: The case of the Swedish Center for Digital Innovation. Communications of the Association for Information Systems. 2021;48(1):31. | Innovation Centre | Sweden | High | Qualitative case study |
| Isabel Jimenez-Zarco, A., M. Cerdan-Chiscano, and J. Torrent-Sellens, Challenges and Opportunities in Science Parks' Management: design of a tool based on the analysis of resident companies. Rbgn-Revista Brasileira De Gestao De Negocios, 2013. 15(48): p. 362-389. | Research parks | Spain | High | Quantitative case study |
| Kepenek, E. B & Eser, Z. 2018. ‘Impact of Pre-incubators on Entrepreneurial Activities in Turkey: Problems, Successes, and Policy Recommendations’ in Innovation and the Entrepreneurial University. New York: Springer. Science, Technology and Innovation Studies (eBook). | Incubators | Turkey | High | Mixed methods cross-sectional |
| Lasrado, V., et al., Do graduated university incubator firms benefit from their relationship with university incubators? Journal of Technology Transfer, 2016. 41(2): p. 205-219. | Incubators | US | Medium | Quantitative longitudinal |
| Lowe N, Schrock G, Jain R, Conway M. Genesis at work: Advancing inclusive innovation through manufacturing extension. Local Economy. 2021 May;36(3):224-41. | Innovation Centre | USA | Low | Qualitative case study |
| Lundqvist, M.A., The importance of surrogate entrepreneurship for incubated Swedish technology ventures. Technovation, 2014. 34(2): p. 93-100. | Incubators | Sweden | High | Mixed methods case study |
| M'Chirgui, Z., et al., University Technology Commercialization through New Venture Projects: An Assessment of the French Regional Incubator Program. Journal of Technology Transfer, 2018. 43(5): p. 1142-1160. | Incubators | France | Unclear | Quantitative longitudinal |
| Merlino, M., et al., INNOVATION PROCESS IN ITALIAN SMES: THE UNIVERSITY ROLE. New Socio-Economic Challenges of Development in Europe 2010. 2011. 120-127. | University | Italy | High | Qualitative case study |
| Naufel, M., THE LUMINOSITY LAB AN INTERDISCIPLINARY MODEL OF DISCOVERY AND INNOVATION FOR THE 21ST CENTURY. Technology and Innovation, 2020. 21(2): p. 115-121. | Labs | US | High | Qualitative case study |
| Nicholls-Nixon, C.L., et al., Entrepreneurial Ecosystems and the Lifecycle of University Business Incubators: An Integrative Case Study. International Entrepreneurship and Management Journal, 2021. 17(2): p. 809-837. | Incubators | Canada | High | Qualitative case study |
| Olcay, G.A. and M. Bulu, Technoparks and Technology Transfer Offices as Drivers of an Innovation Economy: Lessons from Istanbul's Innovation Spaces. Journal of Urban Technology, 2016. 23(1): p. 71-93. | University | Turkey | High | Qualitative case study |
| Pablo-Hernando, S., Technology Centres: An Extended Internal Labour Market for PhD Holders in Spain, in New Voices in Higher Education Research and Scholarship. 2015. p. 107-126. | Innovation Centre | Spain | High | Qualitative cross-sectional |
| Patton, D., Realising potential: The impact of business incubation on the absorptive capacity of new technology-based firms. International Small Business Journal-Researching Entrepreneurship, 2014. 32(8): p. 897-917. | Incubators | UK | High | Qualitative cross-sectional |
| Prokop, V., The Impact of Public Knowledge Investments on Enterprises' Competitiveness: Electronics Industry Case, in Proceedings of the 16th European Conference on Knowledge Management, M. Massaro and A. Garlatti, Editors. 2015. p. 968-975. | Innovation Centre | Czech Republic | High | Quantitative longitudinal |
| Roessner, D., L. Manrique, and J. Park, The economic impact of engineering research centers: preliminary results of a pilot study. Journal of Technology Transfer, 2010. 35(5): p. 475-493. | Research Centres | US | High | Quantitative case studies |
| Roig-Tierno, N., J. Alcazar, and S. Ribeiro-Navarrete, Use of infrastructures to support innovative entrepreneurship and business growth. Journal of Business Research, 2015. 68(11): p. 2290-2294. | University | Spain | Medium | Mixed methods longitudinal |
| Smith, H.L. and S. Bagchi-Sen, Triple helix and regional development: a perspective from Oxfordshire in the UK. Technology Analysis & Strategic Management, 2010. 22(7): p. 805-818. | University | UK | Medium | Quantitative longitudinal |
