# Supplementary material for: The impact of university STEM assets: A systematic review of the empirical evidence
Source: PLoS One. 2023 Jun 28;18(6):e0287005. doi: 10.1371/journal.pone.0287005 (PMC10306183; doi:10.1371/journal.pone.0287005)
Supplement: S1 File — (DOCX) [file pone.0287005.s004.docx]

# **SUPPLEMENT 1**

**Search strategy**

The search strategy was based on a variation of a PICOS statement which is summarised in Table 1.

|  | **Include** | **Exclude** |
| --- | --- | --- |
| **‘Population’** | - Businesses, entrepreneurs, innovators - Intermediaries or professional business services where clearly working with a STEM asset or university. | - Social enterprises and further education providers unless working with a university of STEM asset |
| **‘Interventions’ = STEM Assets** | - Physical STEM assets (incubator, science park, accelerator, research park, laboratories, testing centre, innovation centre) or services provided by STEM asset (business support, technology transfer, training program, testing, information, diagnostics, brokerage, networking) - Programmes delivered by university that support: Innovation (product / process); Knowledge exchange; Cluster development, supply chain; Technical / Degree level apprenticeships; Graduate Programmes and internships | - Generic business support and advice that is not linked to a university or STEM asset. - Start up incubators which little or no focus on innovation and technology transfer or not linked to STEM asset. |
| **‘Outcomes’** | - Innovation capability (process innovation. product innovation, knowledge exchange) regional economic growth (start-ups, cluster development, supply chain), business performance (turnover, sales, productivity, high growth), sustainability, inclusive growth, upskilling and employment, and environment impact. |  |
| **Study Design** | - Any design including ‘policy reviews’; ‘literature reviews’; ‘systematic reviews’; case studies, ‘evaluation’; ‘impact assessment’ ‘value for money studies’ | - Commentaries and editorials, literature reviews, conceptual and theoretical and opinion pieces |
| **Geography** | - OECD member economies i.e. Australia, Austria, Belgium, Canada, Chile, Colombia, Costa Rica, Czech Republic, Denmark, Estonia, Finland, France, German, Greece, Hungary, Iceland, Ireland, Israel, Italy, Japan, Korea, Latvia, Lithuania, Luxembourg, Mexico, Netherlands, New Zealand, Norway, Poland, Portugal, Slovak Republic, Slovenia, Spain, Sweden, Switzerland, Turkey, United Kingdom, United States | - Developing economies |

The search strategy used is shown below, which was adapted to reflect different coverage, indexing terms and volume of literature returned by the three bibliographic search engines. The final Endnote database included results from line 13 of Web of Science (WoS) core collection, line 12 of Econlit and 10 of ERIC resulting in 1,549 returns. Fifteen papers were identified through other sources and after removal of duplicates 1,566 were screened. The Table below presents updated searches undertaken in February 2023.

|  | WoS | Econlit | ERIC |
| --- | --- | --- | --- |
| 1. local firms OR firm* OR SME OR small business OR Large firms OR enterpri* | 463,345 | 290,370 | 27,950 |
| 1. universit* OR HEI) OR higher education institution OR research cent | 915,732 | 154,1430 | 267,981 |
| 1. incubator* OR science park* OR accelerator* OR TOPIC research park* OR lab* OR testing cent* OR innovation cent* | 5,998,099 | 2,999 | 2,190 |
| 1. Intermediaries OR professional services | 112,668 | 8,461 | 2,776 |
| 1. business support OR technology transfer OR training program OR testing OR information OR diagnostics OR brokerage OR networking OR business plan* | 11,617,115 | 205,683 | 250,493 |
| 1. start up OR high growth OR process innovation OR product innovation OR knowledge exchange OR cluster development OR supply chain OR apprenticeship* OR graduate program* OR employ* | 3,121,779 | 17,834 | 3,276 |
| 1. innovation capabilit* OR regional growth OR turnover OR sales OR productivity OR sustainability OR social inclusion OR inclusive growth OR upskilling OR employ* OR GVA) OR gross value add* OR environment OR economic impact* | 2,721,408 | 336,684 | 106,122 |
| 1. #3 AND #2 | 200,309 | 1,086 | 1,540 |
| 1. #8 AND #5 | 136,183 | 353 | 693 |
| 1. #9 AND #1 | 4,213 | 249 | **93*** |
| 1. #7 AND #6 | 1,715,641 | 5,952 | 1,897 |
| 1. #11 AND #10 |  | **85*** | 21 |
| 1. 11 AND #10 AND #9 AND #8 | **1,371*** |  |  |

Searches asterisked included in final databases.
