# Supplementary material for: The impact of university STEM assets: A systematic review of the empirical evidence
Source: PLoS One. 2023 Jun 28;18(6):e0287005. doi: 10.1371/journal.pone.0287005 (PMC10306183; doi:10.1371/journal.pone.0287005)
Supplement: S2 File — (DOCX) [file pone.0287005.s005.docx]

# **Supplement 2**

**Overview of excluded studies**

| **Background (28)** |
| --- |
| 1. Al-Kfairy M, Khaddaj S, Mellor RB. Evaluating the effect of organizational architecture in developing science and technology parks under differing innovation environments. Simulation Modelling Practice and Theory. 2020 Apr;100:102036. |
| 1. Alonso-Conde AB, Rojo-Suarez J, Rentas S. Do Business Administration degrees encourage entrepreneurship and strengthen connection with business incubators? On the Horizon. 2020;28(4)153-163. |
| 1. Breach, A. (2019) Parks and innovation: Lessons from Sheffield’s Advanced Manufacturing Park About Centre for Cities. London. |
| 1. Cadorin E, Johansson SG, Klofsten M. Future Developments for Science Parks: Attracting and Developing Talent. Industry and Higher Education. 2017;31(3)156-167. |
| 1. Caldera A. Debande O. Performance of Spanish universities in technology transfer: An empirical analysis. Research Policy. 2010;39(9)1160-1173. |
| 1. Cattapan P, Passarelli M, Petrone M. Brokerage and SME Innovation: An Analysis of the Technology Transfer Service at Area Science Park, Italy. Industry and Higher Education. 2012;26(5)381-391. |
| 1. Feser D. Innovation intermediaries revised: a systematic literature review on innovation intermediaries’ role for knowledge sharing. Review of Managerial Science. 2022 Oct 8:1-36. |
| 1. Fitjar RD, Rodriguez-Pose A. Firm collaboration and modes of innovation in Norway. Research Policy. 2013;42(1)128-138. |
| 1. Fowlie J, Forder C. Future-proofing placements: The importance of local opportunities for placement students. Higher Education Skills and Work-Based Learning. 2019;9(3)434-444. |
| 1. Franco M, Silva R, Rodrigues M. Partnerships between higher education institutions and firms: The role of students' curricular internships. Industry and Higher Education. 2019;33(3)172-185. |
| 1. Garcia-Alvarez-Coque JM, Mas-Verdu F, Sanchez Garcia M. Determinants of Agri-food Firms' Participation in Public Funded Research and Development. Agribusiness. 2015;31(3)314-329. |
| 1. Hausman N. University innovation and local economic growth. The Review of Economics and Statistics. 2022 Jul 1;104(4):718-35. |
| 1. Jaeger A, Kopper J. Third mission potential in higher education: measuring the regional focus of different types of HEIs. Review of Regional Research. 2014;34(2)95–118. doi:10.1007/s10037-014-0091-3 |
| 1. Jones O, Meckel P, Taylor D. Situated learning in a business incubator: Encouraging students to become real entrepreneurs. Industry and Higher Education. 2021 Aug;35(4)367-383. doi:10.1177/09504222211008117 |
| 1. Kohler T. Corporate accelerators: Building bridges between corporations and startups. Business Horizons. 2016;59(3)347-357. |
| 1. Lackéus M, Williams Middleton K. Venture creation programs: bridging entrepreneurship education and technology transfer. Education and Training. 2015;57(1)48-73. |
| 1. Leung A, et al. Industry Peer Networks (IPNs) Cooperative and competitive interorganizational learning and network outcomes. Management Research Review. 2019;42(1)122-140. |
| 1. Lyu Y, et al. Network embeddedness and inbound open innovation practice: The moderating role of technology cluster. Technological Forecasting and Social Change. 2019 Jul;144:12-24. |
| 1. Martinez Martin, MI, Guillo Rodriguez N, Santero Sanchez R. The Social Economy within Technology Based Entrepreneurship in Spain. A qualitative approach. Ciriec-Espana Revista De Economia Publica Social Y Cooperativa. 2019;96:65-90. |
| 1. Martins JT. Relational Capabilities to Leverage New Knowledge: Managing Directors' Perceptions in UK and Portugal Old Industrial Regions. Learning Organization. 2016;23(6)398-414. |
| 1. Marvel MR, Lumpkin GT. Domain Learning and Opportunity Development in a High-Tech Context. Journal of Enterprising Culture. 2017;25(1)67-96. |
| 1. Morell L. Preparing Global Engineers that Can Develop End-to-End Solutions for Real Business Objectives on the Cloud: a Unique HP-Academia Partnership. ASEE International Forum. Atlanta, Georgia; 2013. doi:10.18260/1-2--17263 |
| 1. Protogerou A, Caloghirou Y, Siokas E. Research networking and technology fusion through EU-funded collaborative projects. Science and Public Policy. 2013;40(5)576-590. |
| 1. Ranga M, Mroczkowski T, Araiso T. University-Industry Cooperation and the Transition to Innovation Ecosystems in Japan. Industry and Higher Education. 2017;31(6)373-387. |
| 1. Sandoval Hamón LA, Ruiz Peñalver SM, Thomas E, Fitjar RD. From high-tech clusters to open innovation ecosystems: a systematic literature review of the relationship between science and technology parks and universities. The Journal of Technology Transfer. 2022 Dec 30:1-26. |
| 1. Sastoque Pinilla L, et al. TRLs 5-7 Advanced Manufacturing Centres. Practical Model to Boost Technology Transfer in Manufacturing. Sustainability. 2019;11(18). |
| 1. Theeranattapong T, Pickernell D, Simms C. Systematic literature review paper: The regional innovation system-university-science park nexus. The Journal of Technology Transfer. 2021 Dec;46(6):2017-50. |
| 1. Theodoraki C, Messeghem K, Rice MP. A Social Capital Approach to the Development of Sustainable Entrepreneurial Ecosystems: An Explorative Study. Small Business Economics. 2018;51(1)153-170. |
|  |
| **Education and training (20)** |
| 1. Alonso-Garcia M., Blazquez-Parra EB, de-Cozar-Macias OD. Planning an industrial design engineering curriculum according to the labour market based on dual training. Journal of Engineering Design. 2020;31(7)399-425. |
| 1. Bone J, Gonzalez-Uribe J, Haley C, et al. The Impact of Business Accelerators and Incubators in The UK. London: Department for Business, Energy & Industrial Strategy; 2019. |
| 1. Business-Higher Education Forum. Forging Strategic Business Partnerships to Develop the 21st Century Workforce: A Case Study of the University of Houston's Undergraduate Petroleum Engineering Program. Washington, DC: Business-Higher Education Forum; 2013. |
| 1. Bartual Figueras MT, Turmo Garuz J. Higher education and employment related skills. The point of view of employers. Revista Complutense De Educacion. 2016;27(3)1211-1228. |
| 1. Gheorghiu A, Treapat L. Liberty Technology Park Cluja Case Study of Successful Entrepreneurship. In: Pînzaru F, Zbuchea A, Brătianu C, Vătămănescu EM, Mitan A, editors. Strategica: International Academic Conference. Shift! Major Challenges of Today's Economy. Bucharest, Romania; 2017 Sep 28-30. p. 551-561. |
| 1. Goncharov VV, Sekerin VD, Akhyadov ESM. Digital Academic Entrepreneurship: New Opportunities for Students In The Development Of The Information Environment. Revista Inclusiones. 2020;7:411-421. |
| 1. Green GM, et al. Recommendations for workforce development in regenerative medicine biomanufacturing. Stem cells translational medicine. 2021 Oct;10(10)1365-1371. |
| 1. Monllau Jaques TM, Rodriguez Avila N. The importance in use of ICT like guarantor agility, efficiency and communications company, university and student. Study case in the faculty of economics and business Universitat Pompeu Fabra. Intangible Capital. 2015;11(4)577-588. |
| 1. Marconi M, Favi C. Eco-design teaching initiative within a manufacturing company based on LCA analysis of company product portfolio. Journal of Cleaner Production. 2020 Jan;242:118424. |
| 1. Leon RD. University - A Knowledge Incubator for Developing Entrepreneurial Skills. In: Zbuchea A, editors. Entrepreneurs. Entrepreneurship: Challenges and Opportunities in the 21st Century. Tritonic: 2017. p. 135-147. |
| 1. Lloyd-Reason L. Entrepreneurship Education: Shifting the Landscape through Embedding Practitioner Experience. In: Kharabsheh RA, editors. Proceedings of the International Conference on Innovation and Entrepreneurship. Amman, Jordan; 2013 Mar 4-5. p. 69-77. |
| 1. Metrolho JC, Ribeiro F, Araujo R. A Strategy for Facing New Employability Trends Using s Low-Code Development Platform. In: Chova LG, Martinez AL, Torres IC, editors. 14th International Technology, Education and Development Conference. Valencia, Spain; 2020 Mar 2-4. p. 8601-8606. |
| 1. National Foundation for Educational Research, London Councils, London Enterprise Panel, South East Strategic Leaders. Improving Employability Skills, Enriching Our Economy. Research Summary. Slough: NFER; 2015 Mar. |
| 1. Pérez-Molina C, Albert Gomez MJ, Gil R, Diaz Orueta G, Sancristobal E, Martin S, et al. Performance-Centered Adaptive Curriculum for Employment Needs. 120th ASEE Annual Conference & Exposition; 2013 Jun 23-26. American Society for Engineering Education; 2013. |
| 1. Portuguez Castro M, Ross Scheede C, Gomez Zermeno MG. The Impact of Higher Education on Entrepreneurship and the Innovation Ecosystem: A Case Study in Mexico. Sustainability. 2019;11(20). |
| 1. Thompson Z, Turula H, Sept D, Brooks SV. The University of Michigan IRACDA Program: Training Future Professors of Engineering and Physiology. Faseb Journal. 2020;34(S1)1. |
| 1. Timmis K, et al. Pipelines for New Chemicals: a strategy to create new value chains and stimulate innovation-based economic revival in Southern European countries. Environmental Microbiology. 2014;16(1)9-18. |
| 1. Suleman F, Laranjeiro AMC. The employability skills of graduates and employers' options in Portugal: An explorative study of anticipative and remedial strategies. Education and Training. 2018;60(9)1097-1111. |
| 1. Wisniewski HS. What is the Business With Ai? Preparing Future Decision Makers And Leaders. Technology and Innovation. 2020 Dec 1;21(4). doi:10.21300/21.4.2020.4 |
| 1. Vaquero-Garcia A, Jesus Ferreiro-Seoane F, Alvarez-Garcia J. Entrepreneurship and University: How to Create Entrepreneurs from University Institutions. In: Peris Ortiz M Alonso Gómez J, Merigó-Lindahl JM, Rueda-Armengot C, editors. Entrepreneurial Universities: Exploring the Academic and Innovative Dimensions of Entrepreneurship in Higher Education. Springer; 2017. p. 47-63. |
|  |
| **No physical STEM asset (17)** |
| 1. Alshaer H, et al. The UK Programmable Fixed and Mobile Internet Infrastructure: Overview, Capabilities and Use Cases Deployment. IEEE Access. 2020;8:175398-175411. |
| 1. Bruno S, Giannoccaro G, Scala ML, Lopopolo G. First activities and power-hardware-in-the-loop tests at the public research laboratory LabZERO. Aeit International Annual Conference. 2018: 1-6. doi:10.23919/AEIT.2018.8577373 |
| 1. Cao L. Data Science: Profession and Education. IEEE Intelligent Systems. 2019;34(5)35-44. |
| 1. Chai S, Freeman RB. Temporary colocation and collaborative discovery: Who confers at conferences. Strategic Management Journal. 2019;40(13)2138-2164. |
| 1. Horgan D, et al. Artificial Intelligence: Power for Civilisation - and for Better Healthcare. Public Health Genomics. 2020;22(5-6)145-161. |
| 1. Ivanova, I., Johnson, M. & Krupenskiy, N. 2018. ‘The Latent Role of Universities in Boosting Innovations: An Informational Approach’ in Innovation and the Entrepreneurial University. New York: Springer. Science, Technology and Innovation Studies (eBook). |
| 1. Kettunen P, Järvinen J, Mikkonen T, Männistö T. Energizing collaborative industry-academia learning: a present case and future visions. European Journal of Futures Research. 2022 Dec;10(1):1-6. |
| 1. Kraatz JA, Hampson KD. Brokering innovation to better leverage R&D investment. Building Research and Information. 2013;41(2)187-197. |
| 1. McBeth CH. Social Innovation in Higher Education: The Emergence and Evolution of Social Impact Centers. Ed.D. Dissertation. 2018, Available from ProQuest LLC. |
| 1. Nicholls-Nixon CL, Valliere D, Singh RM, Hassannezhad Chavoushi Z. How incubation creates value for early-stage entrepreneurs: the People-Place nexus. Entrepreneurship & Regional Development. 2022 Oct 20;34(9-10):868-89. |
| 1. Shvaiko P, Oltramari A, Cuel R, Pozza D, Angelini G. Generating Innovation with Semantically Enabled TasLab Portal. In: Aroyo L, Traverso P, Ciravegna F, Cimiano P, Heath T, Hyvönen E, et al., editors. The Semantic Web: Research and Applications. ESWC 2010. Lecture Notes in Computer Science, vol 6088. Berlin, Heidelberg: Springer; 2010. p. 348-363. doi:10.1007/978-3-642-13486-9_24 |
| 1. Soetanto D, van Geenhuizen M. Life after incubation: The impact of entrepreneurial universities on the long-term performance of their spin-offs. Technological Forecasting and Social Change. 2019;141:263-276. |
| 1. Spithoven A, Vlegels J, Ysebaert W. Commercializing academic research: A social network approach exploring the role of regions and distance. The Journal of Technology Transfer. 2021 Aug;46:1196-231. |
| 1. Taheri M, van Geenhuizen M. Knowledge relationships of university spin-off firms: Contrasting dynamics in global reach. Technological Forecasting and Social Change. 2019;144:193-204. |
| 1. Tavares P, et al. Regional Transformation through Design. 9th International Conference the Future of Education. 2019:421-424. |
| 1. Universities UK. Innovation Vouchers and LEP Structural Funds Strategies. Innovation and Growth Factsheet Series. 2014 Sep;1. |
| 1. Xu D, Nageshwaraniyer SS, Son YJ. A service-oriented simulation integration platform for hierarchical manufacturing planning and control. International Journal of Production Research. 2016;54(23)7212-7230. |
|  |
| **Non-OECD member country (14)** |
| 1. Baskaran A, Chandran VGR, Ng BK. Inclusive Entrepreneurship, Innovation and Sustainable Growth: Role of Business Incubators. Academia and Social Enterprises in Asia. Science Technology and Society. 2019;24(3)385-400. |
| 1. Chen FW, Fu LW, Wang K, T SB, Su CH. The Influence of Entrepreneurship and Social Networks on Economic Growth-From a Sustainable Innovation Perspective. Sustainability. 2018;10(7)2510. |
| 1. Ciobanica ML. Interactive Learning Methods for the Development of Entrepreneurship. In: Roceanu I, et al., editors. Elearning Vision 2020! Vol II. 2016. p. 332-339. |
| 1. Huster K, et al. Global Social Entrepreneurship Competitions: Incubators for Innovations in Global Health? Journal of Management Education. 2017;41(2)249-271. |
| 1. Imanberdiev B, et al. Peculiar features of business incubators functioning: Ukrainian and world experience. Ukrainian Food Journal. 2018;7(2)324-336. |
| 1. Kiran R, Bose SC. Stimulating Business Incubation Performance: Role of Networking, University Linkage and Facilities. Technology Analysis and Strategic Management. 2020;32(12)1407-1421. |
| 1. Lyken-Segosebe D, et al. Stimulating Academic Entrepreneurship through Technology Business Incubation: Lessons for the Incoming Sponsoring University. International Journal of Higher Education. 2020;9(5)1-18. |
| 1. Neamtu DM, Leuciuc GE. Universities and the Potential for the Development of Industrial Clusters in Romania. In: Chova LG, Martinez AL, Torres IC, editors. Edulearn18: 10th International Conference on Education and New Learning Technologies. Palma, Spain; 2018 Jul 2-4. p. 7907-7913. |
| 1. Oh DS, Phillips FE. Technopolis: Best Practices for Science and Technology Cities. New York and Heidelberg: Springer; 2014. |
| 1. Prasetyawan Y, Agustiani E, Jumayla S. Aligning Business Strategy of Incubator Center and Tenants. In: Prasetyo H, et al., editors. Green Process, Material, and Energy: A Sustainable Solution for Climate Change. AIP Conf. Proc. 1855, 030021-1–030021-8; 2017. doi: 10.1063/1.4985491 |
| 1. Rumyantsev AA. Research and Innovation Activity in the Region as a Driver of Its Sustainable Economic Development. Economic and Social Changes-Facts Trends Forecast. 2018;11(2)84-99. |
| 1. Shayakhmetov US, et al. Methodological tools for university transfer of high-demand nanotechnologies to the regional building industry. Nanotechnologies in Construction-a Scientific Internet-Journal. 2021;13(1)12-17. |
| 1. Zhang H, Sonobe T. Business Incubators in China: An Inquiry into the Variables Associated with Incubatee Success. Economics: The Open-Access, Open-Assessment E-Journal. 2011;5. |
| 1. Zhurakovsky VM, Baryshnikova MY, Vorov AB. Modernization of Stem Education in Russia: Traditions and Modern Innovations. Tomsk State University Journal. 2017;(416)87-93. |
|  |
| **Business performance (11)** |
| 1. Barbosa F, Romero F. Evaluation and Adoption of University Technologies by Enterprises. In: Vivas C, Lucas F, editors. Proceedings of the 7th European Conference on Innovation and Entrepreneurship, Vols 1 and 2; Portugal; 2012 Sep 20-21. p. 41-48. |
| 1. Bastianin A, Castelnovo P, Florio M, Giunta A. Big science and innovation: gestation lag from procurement to patents for CERN suppliers. The Journal of Technology Transfer. 2021 Mar 29:1-25. |
| 1. Baviera-Puig A, Buitrago-Vera J, Mas-Verdu F. Trade areas and knowledge-intensive services: the case of a technology centre. Management Decision. 2012;50(7-8)1412-1424. |
| 1. Cadorin E, Klofsten M, Löfsten H. Science Parks, talent attraction and stakeholder involvement: an international study. The Journal of Technology Transfer. 2021 Feb;46:1-28. |
| 1. Campanella F, et al. Quadruple Helix and firms' performance: an empirical verification in Europe. Journal of Technology Transfer. 2017;42(2)267-284. |
| 1. Canovas-Saiz L, March-Chordà I, Yagüe-Perales RM. A quantitative-based model to assess seed accelerators’ performance. Entrepreneurship & Regional Development. 2021 Mar 15;33(3-4):332-52. |
| 1. Ghignoni E, Croce G, Ricci A. Fixed term contracts and employers' human capital: The role of educational spillovers. Papers in Regional Science. 2018;97(2)301-322. |
| 1. Lapinska J, Kadzielawski G. Inter-Organizational Cooperation and Innovative Activity of Enterprises in Poland: Evidence from A Panel Data Analysis. In: Soliman, KS, editors. Vision 2025: Education Excellence and Management of Innovations through Sustainable Economic Competitive Advantage; Madrid, Spain; 2019 Nov 13-14. p. 9046-9053. |
| 1. Laurova M. Competitiveness of Small and Medium Enterprises. In: Rojik S, Pospisil JZ, editors. Proceedings of 8th Annual International Scientific Conference: Competition. 2016. p. 219-229. |
| 1. van Geenhuizen M, Taheri M. Young University Spin-off Firms' Internationalization: The Influence of Founding Teams and Networks. In: Costa C, Au-Yong-Oliveira M, Amorim MPC, editors. Proceedings of the 13th European Conference on Innovation and Entrepreneurship; Aveiro, Portugal; 2018 Sep 20-21. p. 3-11. |
| 1. Vega-Gomez FI, Miranda Gonzalez FJ, Perez-Mayo J. Analyzing the Effects of Institutional-and Ecosystem-Level Variables on University Spin-Off Performance. Sage Open, 2020;10(2)1-14. |
|  |
| **Conceptual, theoretical or discussion paper (7)** |
| 1. Curley MG, Formica P, Nicolo V. From Entrepreneurial Fission to Entrepreneurial Fusion: Achieving Interaction Resonance in a Micro-Innovation Ecology. Industry and Higher Education. 2011;25(1)9-14. |
| 1. Etzkowitz H. Entrepreneurial university icon: Stanford and Silicon Valley as innovation and natural ecosystem. Industry and Higher Education. 2022 Aug;36(4):361-80. |
| 1. Fung HN, Wong CY. Scientific collaboration in indigenous knowledge in context: Insights from publication and co-publication network analysis. Technological Forecasting and Social Change. 2017;117:57-69. |
| 1. Mazzucchelli A, et al. Exploring the microfoundations of innovation capabilities. Evidence from a cross-border R&D partnership. Technological Forecasting and Social Change. 2019;146:242-252. |
| 1. O'Neill M, Scanlan J. Academic innovation: so you want to be a bio-entrepreneur? In: O’Neill M, Hopkins MM, editors. Biotech Manager's Handbook: A Practical Guide. Woodhead Publishing Limited; 2012. p. 23-54. |
| 1. Ranga M, Temel S. From a Nascent to a Mature Regional Innovation System: What Drives the Transition? In: Meissner D, Erdil E, Chataway J, editors. Innovation and the Entrepreneurial University. Science, Technology and Innovation Studies. Springer, Cham; 2018. doi10.1007/978-3-319-62649-9_10 |
| 1. Trueman S, Borrell-Damian L, Smith JH. The Evolution of University-Based Knowledge Transfer Structures. The EUIMA Collaborative Research Project Papers. European University Association: 2014. |
|  |
| **Literature review (3)** |
| 1. Chen JKC, Sun BSS, Batchuluun A. Exploring the Influence Factors for Creation One Knowledge Hub of Science Park: Comparison between Silicon Valley and Hsinchu Science Park. In: Kocaoglu DF, editors. Portland International Conference on Management of Engineering and Technology. 2016 Sep 4-8. p. 1156-1171. |
| 1. Evans N, Bosua R. Knowledge Absorption in Organisations - Development of a Conceptual Process Model. In Vivas C, Sequeira P, editors. Proceedings of the 15th European Conference on Knowledge Management; Santarem, Portugal; 2014 Sep 4-5. p. 321-329. |
| 1. Kanter RM. Enriching the Ecosystem. Harvard Business Review. 2012 Mar;90(3) |
|  |
| **Policy document (2)** |
| 1. Fikirkoca A, Saritas O. Foresight for Science Parks: The Case of Ankara University. Technology Analysis and Strategic Management. 2012;24(10)1071-1085. |
| 1. Zedi I. The Network of Incubators and Clusters in the Republic of Serbia: Improving the Competitiveness of SMEs through Institutional Support. Economic Studies. 2014;23(4)137-158. |
|  |
| **Expert opinion (1)** |
| 1. Plonski GA, et al. Special Session Proposal Performance Indicators for Areas Of Innovation: International Perspective. In: Rafols I, et al., editors. 21st International Conference on Science and Technology Indicators; Valencia, Spain; 2016 Sep 14-16. p. 208-211. |
|  |
| **Unable to obtain (25)** |
| 1. Acosta CE. Experiences in Technological Innovation in The State Of Mexico - Advanced Manufacturing Sector. In: Chova LG, Martinez AL, Torres IC, editors. 7th International Technology, Education and Development Conference; Valencia, Spain; 2013 Mar 4-6. p. 3142-3152. |
| 1. Audretsch D, Lehmann E, Meoli M, Vismara S, editors. University Evolution, Entrepreneurial Activity and Regional Competitiveness Vol. 32. Springer: 2016. |
| 1. Brush CG, Kolvereid L, Widding O, Sørheim R. The Life Cycle of New Ventures: Emergence, Newness and Growth. Cheltenham, UK: Edward Elgar; 2010. |
| 1. Canadian Association of University Teachers. Open for Business: On What Terms? An Analysis of 12 Collaborations between Canadian Universities and Corporations, Donors and Governments. 2013 Nov. |
| 1. Carraquico T, Reis M. Science-To-Business: A Toolkit to Implement Successful Industry (Business)-Academy Partnerships - Case Study On Know Now Know How Network, A Collaboration Experience In Lisbon, Portugal. In: Chova LG, Martinez AL, Torres IC, editors. 13th International Technology, Education and Development Conference; Valencia, Spain; 2019 Mar 11-13. p. 7778-7785. |
| 1. Cavaliere V, Sarti D. Knowledge Sources, Innovation and Organizational Learning in Small Firms. In: Lehner F, Bredl K, editors. Proceedings of the 12th European Conference on Knowledge Management, Vols 1 and 2; Passau, Germany; 2011 Sep 1-2. p. 181-189. |
| 1. Filipe S, Coelho AS, Martins O. Using Complementary Knowledge to Achieve Alternative Results: The Cooperation Between University, Industry and Government. In: Chova LG, Martinez AL, Torres IC, editors. 11th International Conference of Education, Research and Innovation; Seville, Spain; 2018 Nov 12-14. p. 6128-6134. |
| 1. Foncubierta Rodriguez MJ, Perea Vicente JL, Gonzalez Siles G. An Experience in University-Enterprise Relations: The Cogempleo Project In The Technological Foundation Campus Of Algeciras. Educacion Xx1. 2016;19(1)201-225. |
| 1. Fornasari A. The Role of Universities' Third Mission In A Globalized Word: Continuing Education, Social Engagement, Technology Transfer To Relate Science And Society. An International Case Study: If4tm Project. In: Chova LG, Martinez AL, Torres IC, editors. Iceri2016: 9th International Conference of Education, Research and Innovation; Seville, Spain; 2016 Nov 14-16. p. 4373-4379. |
| 1. Gokce N. A New Approach in The Process Of University-Industry Cooperation: The Triple Helix Model. In: Chova LG, Martinez AL, Torres IC, editors. 5th International Conference of Education, Research and Innovation; Madrid, Spain; 2012 Nov 19-21. p. 1156-1156. |
| 1. Guerrero M, Urbano D, Gajon E. Higher Education Entrepreneurial Ecosystems: Exploring the Role of Business Incubators in an Emerging Economy. International Review of Entrepreneurship. 2017;15(2)175-202. |
| 1. Han J, Heshmati A. Determinants of Financial Rewards From Industry-University Collaboration In South Korea. International Journal of Innovation Management. 2016;20(7). |
| 1. Heim D, Fischer M, Winkelmann A. Improving Concepts of E-Learning by Using ERP Systems for an Interactive Knowledge Diffusion. In: Zaphiris P, Ioannou A, editors. Learning and Collaboration Technologies: Novel Learning Ecosystems Lct 2017, Pt I; Vancouver, BC; 2017 Jul 9-14. p. 199-215. |
| 1. Jakubiak M, Chrapowicki P. Scientists' Entrepreneurial Competencies as Determinants of University-Industry Cooperation. In: Chova LG, Martinez AL, Torres IC, editors. Edulearn18: 10th International Conference on Education and New Learning Technologies; Palma, Spain; 2018 Jul 2-4. p. 10567-10572. |
| 1. Jesus Luengo-Valderrey M. Impact of the Triple Helix and the Difficulties to Innovate in the Innovation Aims: Spain, 2007-2013. Revista De Estudios Regionales. 2018;(113)165-192. |
| 1. Karlsson C, Johansson B, Stough RRE. Entrepreneurship, Social Capital and Governance: Directions for the Sustainable Development and Competitiveness of Regions. Cheltenham, UK: Edward Elgar; 2012. |
| 1. Kingma B. Academic Entrepreneurship and Community Engagement: Scholarship in Action and the Syracuse Miracle. Cheltenham, UK: Edward Elgar; 2011. |
| 1. Lofsten H. Critical Resource Dimensions for Development Of Patents - An Analysis Of 131 New Technology-Based Firms Localised In Incubators. International Journal of Innovation Management. 2015; 19(1)1550006. |
| 1. Novotny A, Rasmussen E, Clausen TH, Wiklund J. Research Handbook on Start-Up Incubation Ecosystems. Cheltenham, UK: Edward Elgar; 2020. |
| 1. Pablo-Hernando S. Transferring knowledge: PhD holders employed in Spanish technology centres. International Journal of Technology Management. 2015;68(3-4)228-254. |
| 1. Palmieri S, Amandolese D. Innovation and Competitiveness Go Via Universities. In: Chova LG, Martinez AL, Torres IC, editors. Inted2017: 11th International Technology, Education and Development Conference; Valencia, Spain; 2017 Mar 6-8. p. 3081-3089. |
| 1. Palmieri S, Amandolese D. Universities and Industry: Networks To Co-Innovate. In: Chova LG, Martinez AL, Torres IC, editors. Iceri2016: 9th International Conference of Education, Research and Innovation. 2016. p. 6648-6658. |
| 1. Pena Aguilar JM, et al. Innovation in Developing New Products in Collaboration Academia Industry. Case Study: Chocolate Coating. In: Chova LG, Martinez AL, Torres IC, editors. Edulearn15: 7th International Conference on Education and New Learning Technologies; Barcelona, Spain; 2015 Jul 6-8. p. 5553-5559. |
| 1. Remdisch S, et al. Gaining Regional Competitiveness by Increasing Employer Attractiveness in Small and Medium-Sized Enterprises: New Approach for Knowledge Transfer Between Academia and Business. In: Audretsch D, Lehmann E, Meoli M, Vismara S, editors. University Evolution, Entrepreneurial Activity and Regional Competitiveness Vol. 32. Springer: 2016. p. 175-191. |
| 1. Stejskal J, Hajek P. Does University-Industry Cooperation Lead to Innovation? - Case Study of Czech Republic. In: Chova LG, Martinez AL, Torres IC, editors. Iceri2015: 8th International Conference of Education, Research and Innovation; Seville, Spain; 2015 Nov 18-20. p. 6659-6669. |
